# Implications of mtDNA in human health and diseases

**DOI:** 10.5114/bta/204532

**Published:** 2025-06-30

**Authors:** Smruthi Seethashankar, Shruti Hariharan, Venkatachalam Deepa Parvathi

**Affiliations:** 1 Department of Biomedical Sciences, Sri Ramachandra Institute of Higher Education and Research, Chennai, India; 2 Amity Institute of Molecular Medicine and Stem Cell Research, Amity University, Uttar Pradesh, India

**Keywords:** mtDNA, mtDNA CN, mitochondrial diseases, cancer, neurodegenerative diseases, cardiovascular diseases, renal diseases, fertility

## Abstract

The maternally inherited autonomous organelles, mitochondria, are responsible for a myriad of functions within the cell. They may contain more than one copy of DNA and can themselves be present in multiple numbers within a cell. The integrity of the mitochondrial genome is affected by variations in DNA copy number or the presence of mutations. Compromising this integrity has been documented to result in disorders affecting various systems. Focusing on such trends could enhance knowledge essential for developing strategies to manage these disorders. Irregular patterns of mitochondrial DNA (mtDNA) copy number (CN) variation have been identified in various cancers. Reduced mtDNA CN has been associated with neurodegenerative disorders, cardiovascular diseases, and kidney disorders. Mutations in the mitochondrial respiratory chain complex have been linked to cardiomyopathy. High rates of mtDNA deletions have been found in aging patients and subjects with Parkinson’s disease. While sperm function appears to deteriorate with increased mtDNA CN, oogenesis involves a significant increase to enable the oocyte to achieve fertilization and further development. Prospective therapies to treat mitochondrial diseases may include approaches that aim to reduce the levels of mutant mtDNA below the disease-causing threshold, such as targeted removal of defective mitochondria. Mutations in mitochondrial DNA contribute to various diseases; some single substitutions appear to disrupt the normalcy of more than one organ, underscoring the importance of mitochondrial genome integrity. The presence of mutations and copy number variations may serve as diagnostic markers and also provide insight into prognosis.

## Introduction

Mitochondria, the autonomous organelles of the cell, contain their DNA (termed mitochondrial DNA or mtDNA), which encodes 13 vital respiratory chain proteins. Given that mitochondria are integral to the cell’s bioenergetics, any qualitative or quantitative alterations in the mitochondrial genome can disrupt normal cellular functioning.

mtDNA is inherited exclusively from the mother (Giles et al. 1980), as paternal mitochondria are eliminated from the cells post-fertilisation (Kaneda et al. 1995). Additionally, mtDNA does not undergo recombination; hence, any mtDNA polymorphisms are inherited as such from the maternal line (Bray and Ballinger 2017).

Generally, the mitochondria within a cell share identical DNA content, a condition referred to as homoplasmy. In cases where certain mtDNA sequences are altered due to mutations, a combination of wild-type and mutant mtDNA may coexist within the same cell, a condition known as heteroplasmy. Reversion from heteroplasmy to homoplasmy can occur within a few generations (Santos et al. 2006).

**Figure 1:**
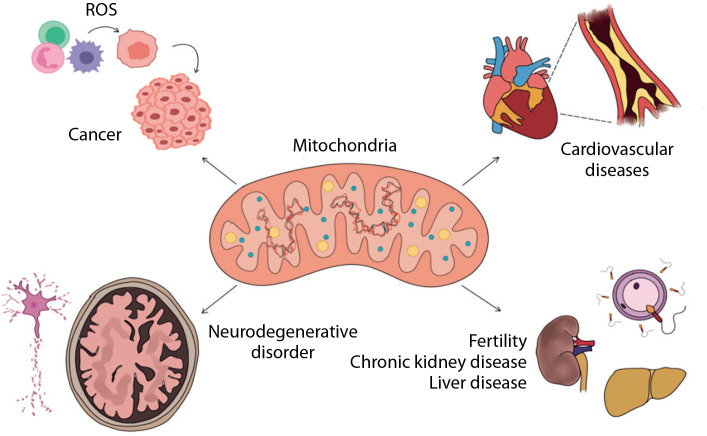
Systemic conditions that arise as a result of mitochondrial DNA mutations and copy number variations

Beyond their bioenergetic functions, mitochondria also influence the cellular phenotype by regulating mRNA abundance, translation, and alternative splicing (Guantes et al. 2015). They are responsible for regulating approximately half of the total protein levels in a cell, owing to dosage effects on transcription and the content and function of the translation apparatus. Maintaining the integrity of the mitochondrial genome is crucial, as even single-base substitutions have been linked to disorders affecting multiple organ systems (Stewart and Chinnery 2015). It is essential to understand the mutations and their resultant effects, enabling the betterment of diagnosis and treatments available. mtDNA mutations and their manifestations as disorders such as cancer, cardiovascular disease (CVD), and neurodegenerative disorder among others are elucidated in this review (Figure 1), followed by the possible treatment modalities.

## mtDNA CN and mutations in mtDNA

mtDNA CN varies across different tissues and developmental stages, and this variation is regulated through mitochondrial fission and fusion (Castellani et al. 2020). The regulation of mtDNA CN has been hypothesized to occur via a threshold mechanism, where a low threshold triggers replication and a high threshold induces degradation. Although mtDNA replication is not synchronous with that of nuclear DNA (nDNA), it is largely dependent on nDNA-encoded factors, such as trans-acting elements, that regulate mtDNA CN. While mtDNA CN does not provide direct information about mtDNA damage, it is associated with mitochondrial enzyme activity and ATP production and can therefore serve as a biomarker for mitochondrial function (Ashar et al. 2017; Yiyi Zhang et al. 2017).

**Figure 2:**
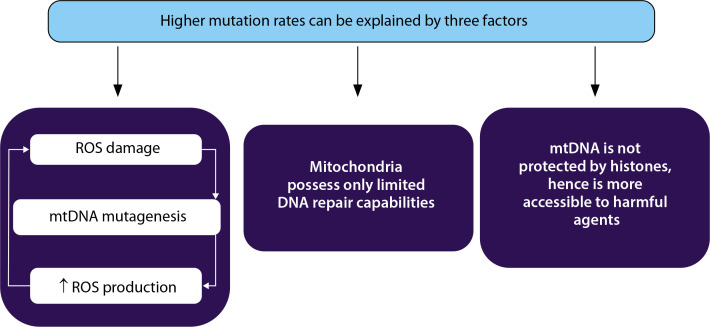
High mutation rate seen in mitochondrial DNA (mtDNA) can be explained by three factors. First, mtDNA is located in the mitochondrial matrix near the electron transport system where reactive oxygen species (ROS) are continuously generated. Second, mtDNA is not protected by histones and chromatin structure, and third, mitochondria possess only limited DNA repair capabilities (Fang et al. 2004)

Mutations in mtDNA, particularly in critical regions such as promoters, transcription factor binding sites, and replication origins, can lead to reduced gene expression and impaired mitochondrial biogenesis. These mutations can also affect mitochondrial respiration capacity and increase the rate of apoptosis (Figure 2).

**Figure 3:**
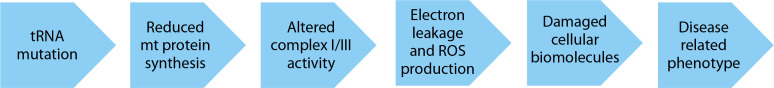
Mitochondrial DNA (mtDNA) mutations commonly seen in cardiovascular diseases have different mechanisms of action: (1) mutations in transfer RNA (tRNA); (2) mutations in the OXPHOS components significantly reduce ATP synthesis and result in increased ROS production; and (3) mutations in the D-loop disrupt the normal mtDNA replication process and decrease mtDNA copy number (Dabravolski et al. 2022)

Mitochondrial malfunction, most commonly due to a loss of efficiency in oxidative phosphorylation resulting from the defective synthesis of mitochondrial ribosomal proteins, has been linked to cancer, CVD, neurodegeneration, diabetes, muscle atrophy, aging, and other age-associated human conditions (Chen et al. 2023). This may be attributed to the fact that mitochondrial dysfunction results in inefficient energy production, elevated levels of reactive oxygen species (ROS) that can damage lipids, proteins, and nucleic acids, and altered expression of nuclear genes involved in metabolism, growth, differentiation, and apoptosis (Castellani et al. 2020) (Figure 3).

The distribution of mutant mtDNA can differ among tissues and organs due to factors such as mutation load, replication dynamics, and tissue-specific energy requirements. Consequently, the severity and manifestation of mitochondrial mutations can vary across affected organs (Stewart and Chinnery 2015). Discussed below are two mutations that occur in different body systems, leading to various diseases and conditions.

The m.3243A>G mutation in the *MT-TL1* gene (tRNA-Leu) can simultaneously affect the neurological, cardiovascular, and renal systems. This mutation is commonly associated with MELAS syndrome and can impair mitochondrial function and energy production, leading to a range of symptoms and multiorgan involvement (Sproule and Kaufmann 2008). Individuals carrying this mutation may experience stroke-like episodes, which manifest as temporary neurological deficits such as weakness, numbness, or paralysis. Less commonly, they may also develop encephalopathy, characterized by cognitive impairment, seizures, migraines, and ataxia (Nesbitt et al. 2013).

The m.3243A>G mutation can also result in various cardiac manifestations, including cardiomyopathy, arrhythmias, and conduction abnormalities. It is notably associated with hypertrophic cardiomyopathy, which is characterized by abnormal thickening of the heart muscle. Although rare, kidney complications may occur in MELAS syndrome. This mutation can lead to renal tubular dysfunction, impairing the kidneys’ ability to reabsorb and excrete substances properly. Such dysfunction may present as renal tubular acidosis, aminoaciduria, or renal insufficiency (El-Hattab et al. 2015).

The m.8344A>G mutation in the *MT-TK* gene, which involves tRNA-Lys, is another mtDNA mutation that can affect multiple organ systems. It is associated with a group of disorders known as myoclonic epilepsy with ragged red fibers (MERRF) syndrome, so named because the affected muscle fibers contain accumulations of defective mitochondria. Individuals with this mutation may exhibit muscle weakness, exercise intolerance, and myopathy, which can affect both voluntary muscles and muscles involved in vital functions such as breathing (Enriquez et al. 1995).

The m.8344A>G mutation primarily impacts the central nervous system. Individuals with MERRF syndrome may suffer from myoclonus, epileptic seizures, and progressive ataxia. They may also develop cognitive impairment, dementia, and other neurological symptoms (Ripolone et al. 2023). Visual impairments are also common in MERRF syndrome and may include retinal pigmentary changes, optic atrophy, and progressive vision loss. Some individuals may also develop cataracts (Al-Enezi et al. 2008).

Random segregation of mitochondria during mitosis in heteroplasmic cells can influence the proportion of mutant mtDNA in the daughter cells. Clinical expression of the mutation typically occurs when the level of mutant mtDNA exceeds the tissue’s pathogenic threshold (Table 1). Loss of mutant mtDNA may also occur in rapidly dividing tissues. Generally, a cell requires about 60–90% mutant mtDNA for diseaselike manifestations to appear in the affected tissue (Tuppen et al. 2010). However, in rare cases – such as the m.5545C>T mutation – less than 25% mutant mtDNA has been found to cause tissue damage, suggesting that some mitochondrial mutations may act dominantly, contrary to their typical recessive behavior (Sacconi et al. 2008).

**Table 1:** Point mutations (substitutions) found to occur in mitochondrial DNA and the resulting conditions

Point mutation	Disease condition	Reference
m.3243 A>G	MELAS syndrome	Sproule and Kaufmann 2008
m.8344 A>G	MERRF syndrome	Enriquez et al. 1995
m.8993 T>G/C	Neuropathy, ataxia and retinitis pigmentosa (NARP) syndrome	Sproule and Kaufmann 2008
m.5545 C>T	Multisystemic disorder	Sacconi et al. 2008
m.14709 T>C	Myopathy, weakness, diabetes	Mancuso et al. 2005
m.4263 A>G	Hypertension	Wang et al. 2011

## Mitochondrial dynamics

Mitochondrial dynamics encompass the continuous processes of fusion and fission that the organelle undergoes, enabling it to respond to cellular demands and maintain functionality. Mitochondrial fission plays a crucial role in initiating mtDNA replication, while mitochondrial fusion is essential for the proper distribution of the replication machinery throughout the mitochondrial network. Maintaining a balance between these two processes is vital for preserving the integrity and CN of mtDNA (Sabouny and Shutt 2021).

The key proteins regulating mitochondrial fission and fusion in mammals include dynamin-related protein 1 (DRP1), mitofusins 1 and 2 (MFN1/2), and the dynaminlike GTPase optic atrophy 1 (OPA1). MFN1, MFN2, and OPA1 facilitate mitochondrial fusion, whereas DRP1 is primarily responsible for mitochondrial fission (Laaper and Jahani-Asl 2018). Impairments in the proteins that regulate mitochondrial dynamics can result in disease, commonly manifesting as neuromuscular and neurodegenerative conditions (Dorn and Dang 2022).

Homozygous OPA1 mutant mice do not survive past embryonic development, while heterozygous OPA1 mutants exhibit key features of human dominant optic atrophy. These features include abnormal mitochondrial morphology, disorganized cristae structure, mitochondrial dysfunction, and mtDNA instability. A significant decrease in mtDNA copy number has been observed in hearts with OPA1 mutations, correlating with the mitochondrial dysfunction reported. The loss of mtDNA integrity is believed to play a critical role in the cardiac abnormalities associated with these OPA1 mutations (Alavi et al. 2007; Chen et al. 2012).

Mitochondrial fission can lead to heightened production of ROS. When fusion proteins such as MFN1/2 are reduced, mitochondria become fragmented, resulting in elevated ROS levels and creating a self-reinforcing cycle of oxidative stress. Conversely, mitochondrial fusion plays a crucial role in reducing excessive ROS by maintaining a connected mitochondrial network. Disruption of these processes can further escalate ROS production, compounding mitochondrial dysfunction.

The efficiency of oxidative phosphorylation (OXPHOS) is directly influenced by mitochondrial dynamics. Fragmented mitochondria often exhibit a loss of mitochondrial membrane potential (∆Ψm) and decreased ATP production, resulting in inefficient OXPHOS. This inefficiency can exacerbate oxidative stress and may shift energy production from OXPHOS to glycolysis, a pattern frequently observed in cancer cells. MtDNA integrity is closely related to mitochondrial dynamics. Fragmented mitochondria can suffer from increased oxidative damage to mtDNA, potentially causing mutations that impair mitochondrial function. This damage is linked to various diseases, including cancer, where mitochondrial dysfunction is a hallmark (Ježek et al. 2018; Kim and Song 2016; Kondadi et al. 2019).

## Cancer

mtDNA mutations and copy number variations have been implicated in various cancer types (Table 2). In tumors, mitochondria can undergo changes that are rarely observed in their normal counterparts, and these alterations may contribute to tumor development. Such changes include the Warburg effect – a metabolic shift characterized by increased glucose uptake and fermentation of glucose to lactate despite the presence of oxygen (Liberti and Locasale 2016) – dysfunction of key mitochondrial enzymes such as succinate dehydrogenase and fumarate hydratase (King et al. 2006), and altered functioning of the electron transport chain, which can lead to excessive production of ROS (Ishikawa et al. 2008).

**Table 2:** Trend of mitochondrial DNA (mtDNA) copy number (CN) across various cancers and other conditions

Disease condition	Trend of mtDNA CN	Reference
**Cancer**		
Oncocytoma	Increase	Tickoo et al. 2000
Breast cancer	Decrease	Mambo et al. 2005; Tseng et al. 2006
Hepatocellular carcinoma	Decrease	Lee et al. 2004
Clear cell renal cell carcinoma	Decrease Increase	Meierhofer et al. 2004; Mambo et al. 2005
Ovarian cancer	Increase	Meierhofer et al. 2004
Gastric cancer	Decrease	Wu et al. 2005
Bladder cancer	Decrease	Reznik et al. 2016
Oesophagus cancer	Decrease	Mondal et al. 2013
Head and neck squamous cell cancer	Decrease	Reznik et al. 2016
Lung adenocarcinoma	Decrease	Reznik et al. 2016
Oral cancer	Decrease	Mondal et al. 2013
Colorectal cancer	Increase Decrease	Feng et al. 2011; Cui et al. 2013
Cervical cancer	Increase	Sun et al. 2020
Prostate cancer	Increase Decrease	Tu et al. 2015; Moore et al. 2017
Glioblastoma	Increase	Radzak et al. 2022
Leukaemia	Increase	Lan et al. 2008
Melanoma	Increase	Radzak et al. 2022
Pancreatic cancer	Decrease	Tuchalska-Czuroñ et al. 2019
**Neurodegenerative diseases**		
Alzheimer’s disease	Decrease	Liou et al. 2021
Parkinson’s disease	Increase	Bury et al. 2017
Amyotrophic lateral sclerosis	Increase	Harvey et al. 2024
**Cardiovascular diseases**		
Cardiac arrhythmias, hypertension, hyperlipidemia	Decrease	Foote et al. 2018
**Renal disease**		
Chronic kidney disease, end stage renal disease	Increase	Johnson et al. 2023

Alterations in mtDNA levels have been observed in several cancer types, although there is ambiguity regarding the direction of these changes. mtDNA CN is decreased in bladder, breast, esophageal, head and neck squamous, kidney, and liver cancers. However, at least one sample in each of these cancer types showed an increase in mtDNA CN, contrary to the overall trend. In contrast, lung adenocarcinoma demonstrated a higher rate of mtDNA accumulation compared to normal tissue.

mtDNA CN has also been investigated for its potential as a prognostic marker. However, its association with patient survival varies across cancer types. High mtDNA levels have been linked to better survival in adrenocortical carcinoma, chromophobe renal carcinoma, and low-grade glioma, whereas poorer survival outcomes were associated with clear-cell renal cell carcinoma and melanoma (Reznik et al. 2016).

Somatic mtDNA substitutions are most frequent in gastric, hepatocellular, colorectal, and prostate cancers, and are relatively fewer in hematologic cancers. These variations may arise due to multiple factors, such as differences in mutation rates among cell lineages or the influence of selection pressure determining the number of mutations (Ju et al. 2014). Germline mutations in mtDNA at positions 10,398 and 16,198 have been linked to both breast and endometrial cancers. Somatic mutations in tumor mtDNA range from major insertion–deletion events and chain-termination changes to more subtle missense mutations (Brandon et al. 2006). mtDNA CN is significantly lower in highgrade tumors compared to low-grade tumors (Wang et al. 2006). However, any information on the spatial variation of mtDNA CN across a tumor – if such variation exists – has yet to be elucidated.

Mitochondrial mutations can occur during tumor evolution and may progress toward a nearly homoplasmic condition. The mutation rate and cell proliferation rate are linearly correlated with the drift of mitochondrial mutations toward homoplasmy (Ju et al. 2014). The somatic mutation rate of mtDNA is further amplified by increased ROS levels during neoplastic transformation (Penta et al. 2001).

Recurrent mutations associated with cancer have been found at 246 mtDNA positions, a recurrence rate 6.9 times higher than expected by chance. This suggests that the generation or fixation of mtDNA mutations does not occur randomly but is instead influenced by factors such as positive selection or underlying mutational processes. In samples containing multiple substitutions, more than half of the mutations were found to be linked within the same mtDNA genome. Reinforcing the nonrandom nature of these events is the excessive mtDNA CN per cell.

A total of 1907 mtDNA substitutions have been reported in cancer samples, with the number of mutations varying by cancer type. For instance, gastric, hepatocellular, and prostate cancers exhibit a higher number of mtDNA mutations, whereas hematologic cancers display fewer. While most of these mutation events appear to be non-specific, the observed disparities in mtDNA CN trends across cancers may be attributed to factors such as different cell lineages or their origin from distinct mitochondrial genome generations, among others (Ju et al. 2014).

## Neurodegenerative diseases

Neurodegenerative diseases are generally associated with reduced levels of mtDNA CN. The accumulation of mtDNA mutations can impair the function of electron transport chain complexes, thereby reducing ATP production and increasing ROS formation. Conversely, increased ROS levels can induce the accumulation of new mtDNA mutations, forming a feedback loop between mtDNA mutations and ROS generation that contributes to cell death. In many neurodegenerative disorders, including Alzheimer’s disease, Parkinson’s disease, and amyotrophic lateral sclerosis, mtDNA D-loop methylation is inversely associated with mtDNA CN, highlighting its importance in disease progression (Coppedè and Stoccoro 2019). These conditions and their associated mtDNA abnormalities are described in Table 3.

**Table 3:** Neurodegenerative diseases and the associated abnormality in mitochondrial DNA (mtDNA)

Disease	mtDNA abnormality	Description	Reference
Alzheimer’s disease	Reduced mtDNA CN, mitochondrial structural changes	Reduced mtDNA CN and altered mitochondrial morphology contribute to neurodegeneration	Nissanka and Moraes 2018
Parkinson’s disease	Increased mtDNA deletions, cytochrome c oxidase deficiency, reduced mtDNA CN	mtDNA deletions impair mitochondrial function, also observed in peripheral blood as a potential biomarker	Pyle et al. 2015
Amyotrophic lateral sclerosis	Reduced mtDNA CN, D-loop methylation inversely related to mtDNA CN	mtDNA depletion correlates with disease progression and mitochondrial dysfunction in motor neurons	Coppedè and Stoccoro 2019
Creutzfeldt-Jakob disease	Reduced mtDNA CN	mtDNA depletion contributes to mitochondrial dysfunction and neurodegeneration	Wei et al. 2017
Huntington’s disease	Altered mitochondrial morphology, dysregulated fission/fusion gene expression	Mutant huntingtin disrupts mitochondrial function, affecting energy production and neuronal survival	Shirendeb et al. 2011
NARP	T8993G/C mutation in ATPase 6 gene	Point mutation impairs ATP production, causing neuropathy, ataxia, and retinitis pigmentosa	Sproule and Kaufmann 2008
MELAS	A3243G mutation in tRNA leucine gene	Mutation in the tRNA gene disrupts mitochondrial protein synthesis, leading to various neurological symptoms	Sproule and Kaufmann 2008
Alpers-Huttenlocher syndrome	*POLG* mutation	*POLG* mutation impairs mtDNA replication, leading to progressive neurodegeneration and liver dysfunction	Pinto and Moraes 2014
Ataxia neuropathy spectrum	*POLG* mutation	*POLG* mutations cause mtDNA replication defects, leading to ataxia and neuropathy	Pinto and Moraes 2014
Childhood myopathy, encephalopathy, and ophthalmoplegia	*POLG* mutation	*POLG* mutations impair mtDNA replication, resulting in muscle weakness and neurological deterioration	Pinto and Moraes 2014

Neurons and myocytes, which become non-proliferative post-mitotically, are particularly affected by mtDNA alterations. Due to their inability to divide, it is difficult to eliminate cells carrying extensive damage or high levels of mtDNA mutations (Pinto and Moraes 2014), a situation that develops progressively with age (Santos et al. 2010).

Age-related neurodegenerative disorders often involve structural mitochondrial alterations. For example, in Alzheimer’s disease, the mitochondrial number is reduced, while the mitochondrial size is increased, and inner membrane cristae are disrupted (Nissanka and Moraes 2018). In Parkinson’s disease, elevated rates of mtDNA deletions result in cytochrome c oxidase deficiency in neurons, along with region-specific reductions in mtDNA levels in affected brain areas. This reduction can also be detected in peripheral blood, suggesting its potential as a biomarker for Parkinson’s diagnosis. A similar decline in mtDNA CN has been observed in Alzheimer’s and Creutzfeldt-Jakob diseases (Pyle et al. 2015; Wei et al. 2017).

Mutations in nuclear genes responsible for preserving mtDNA integrity, such as POLG and PEO1, can disrupt mtDNA replication and further contribute to mitochondrial dysfunction. These mutations are linked to various syndromes, including Alpers-Huttenlocher syndrome, ataxia neuropathy spectrum, and childhood myo-cerebro-hepatopathy with ophthalmoplegia (Pinto and Moraes 2014).

Certain mitochondrial diseases, including neuropathy, ataxia, retinitis pigmentosa (NARP), and MELAS, result from specific point mutations in mtDNA-encoded genes. For instance, the T8993G/C mutation in the ATPase 6 gene is frequently associated with NARP, while the A3243G mutation in the tRNA leucine gene is commonly found in MELAS (Sproule and Kaufmann 2008).

In Huntington’s disease, the mutant huntingtin gene disrupts mitochondrial metabolism, affecting neuronal survival and altering mitochondrial localization and morphology. In affected individuals, mitochondrial fission genes are upregulated, whereas fusion genes are expressed at lower levels. Additionally, genes related to mitochondrial complexes I, III, IV, and V are overexpressed, possibly as a compensatory mechanism to counteract the mitochondrial dysfunction induced by the huntingtin mutation (Shirendeb et al. 2011).

## Cardiovascular diseases

In any CVD, mtDNA CN is inversely proportional to the risk and occurrence of the condition. A decrease in mtDNA CN in myocardial cells can lead to mitochondrial dysfunction, impairing ATP production and energy supply to ion channels, thereby disrupting ion homeostasis and leading to cardiac arrhythmias. An increase in ROS beyond a certain threshold can trigger mitochondrial ROS-induced ROS release, which causes mitochondrial damage through oxidative stress, further contributing to reduced mtDNA CN (Yiyi Zhang et al. 2017).

Low levels of mtDNA have been shown to indicate a high risk of CVD and an increased likelihood of sudden cardiac death (Yue et al. 2018). As mtDNA CN is inversely associated with the incidence and prevalence of type 2 diabetes mellitus (T2DM), it may also mediate the relationship between CVD and T2DM. Heart tissue samples from patients with coronary artery disease (CAD) have demonstrated significant mtDNA deletions (Memon et al. 2021). Lower mtDNA CN has also been linked to cardio-metabolic traits and agerelated conditions independently associated with obesity, hypertension, diabetes, and hyperlipidemia (Liu et al. 2021).

Measuring mtDNA levels in the myocardium can be done indirectly through mtDNA levels in peripheral blood, as a positive correlation has been established between the two (Knez et al. 2017).

Low-density lipoprotein (LDL) appears to exert a causal effect on mtDNA CN; however, the reverse – an effect of mtDNA CN on LDL – has not been observed. Since LDL is a known causal factor for coronary heart disease (CHD), CVD may, in part, result from the influence of elevated LDL levels on reduced mtDNA CN (Liu et al. 2023).

Mitochondrial malfunction is also an early event in the progression of atherosclerosis. Disruptions in mitochondrial homeostasis lead to increased ROS production, which impairs calcium metabolism and reduces energy production. Elevated ROS levels play a critical role in the onset of cardiovascular complications by promoting the proliferation and migration of vascular smooth muscle cells, triggering inflammatory responses, and increasing free calcium levels in endothelial cells. In cardiomyocytes, high levels of mitochondrial ROS can result in mtDNA loss, increased autophagy, and altered calcium homeostasis, ultimately affecting mitochondrial dynamics, morphology, and function (Fang et al. 2004).

Studies have identified mtDNA mutations in respiratory chain complexes I, III, and IV, as well as in mitochondrial 16S and 12S rRNAs, in patients with cardiomyopathy (Bray and Ballinger 2017). Additionally, multiple mtDNA deletions with autosomal dominant inheritance have also been associated with cardiomyopathy (Majamaa-Voltti et al. 2002).

## Renal diseases

Dysfunctional mitochondria in kidney cells have been associated with the development of chronic kidney disease (CKD). Increased levels of mtDNA in peripheral blood have been linked to a lower risk of developing the condition, which is further supported by evidence showing that higher mtDNA levels are associated with a reduced likelihood of developing diabetes and microalbuminuria, both of which are risk factors for CKD (Tin et al. 2016). In patients with end-stage renal disease (ESRD), cell-free mtDNA levels were found to be elevated, while intracellular mtDNA levels were decreased compared to healthy individuals (Yuheng Zhang et al. 2017).

Mitochondrial damage and resulting dysfunction are also implicated in the pathophysiology of acute kidney injury (AKI). While most renal mitochondrial diseases primarily cause tubular damage, mutations in the coenzyme Q10 biosynthesis pathway and mtDNA 3243 mutations are more commonly associated with glomerular diseases (Connor et al. 2017).

The progression of renal disorders is influenced by mitochondrial dysfunction resulting from mtDNA mutation, mtDNA leakage, and mtDNA methylation. Leaked mtDNA that accumulates in the cytoplasm is recognized as an endogenous pathogen, triggering inflammatory and innate immune responses through multiple signaling pathways. The levels of mtDNA in peripheral serum and urine are known to reflect the extent of kidney injury (Feng et al. 2022).

## mtDNA: key to fertility and reproductive health

There are approximately 100 copies of mtDNA in sperm, whereas mature oocytes contain over 150,000 copies, a number significantly higher than that found in most somatic cells. Downregulation of mtDNA copy number is necessary for normal sperm function, whereas a high mtDNA copy number is required during oogenesis to ensure proper distribution of mitochondria and other organelles to the cells of the early postimplantation embryo, before mitochondrial biogenesis and mtDNA replication resume.

A fundamental aspect of the oocyte is that its growth is dependent on mitochondrial biogenesis and mtDNA replication (Cummins 1998). As an oocyte progresses from the primordial follicle stage to a mature follicle, there is a significant increase in mtDNA copy number. This clonal expansion of mtDNA halts when the oocyte reaches metaphase II, and after fertilization, mtDNA CN does not increase further. Cell division in early embryos occurs without accompanying mitochondrial replication, resulting in blastomeres formed during cleavage containing progressively fewer mtDNA copies until implantation. Consequently, oocytes with low mtDNA CN exhibit reduced viability and fertilizability (Santos et al. 2006).

With age, the mitochondrial genome deteriorates due to accumulating point mutations and rearrangements (Jansen and De Boer 1998), and it is known that the mitochondrial genome accumulates mutations at a rate seventeen times higher than that of the nuclear genome (Wallace et al. 1987). As a result, there appears to be a need for female sterility before complete oocyte depletion, a phenomenon termed oopause (Jansen and De Boer 1998).

Immature oocytes also show lower fertilization rates compared to oocytes that have achieved cytoplasmic maturity, which may, in part, be governed by mitochondrial biogenesis. Therefore, failure of *in vitro* fertilisation in some cases could be attributed to immature oocytes resulting from defective mitochondrial biogenesis (Reynier et al. 2001). Ovarian insufficiency has also been characterized by low mtDNA content (May-Panloup et al. 2005), and mitochondrial mutations have been identified in over 40% of women experiencing infertility due to poor ova quality (Shamsi et al. 2013).

While mtDNA CN increases during oogenesis, a rapid reduction is observed during spermatogenesis. mtDNA CN in men with abnormal semen is significantly higher than in those with normal semen (May-Panloup et al. 2003; Popova et al. 2022). This increase may be due to: (i) excessive ROS production by infertile individuals, which can alter sperm mtDNA CN; (ii) a feedback response attempting to compensate for fragmented or mutated mtDNA within defective mitochondria; (iii) abnormal spermatogenesis, which can lead to increased mtDNA CN in infertile men. In addition to higher mtDNA CN, a decrease in mtDNA integrity has also been observed in individuals with sperm abnormalities (Song and Lewis 2008). mtDNA deletions are more frequent in individuals with asthenozoospermia compared to unaffected individuals (Kao et al. 1995). Moreover, the activity of mitochondria-encoded respiratory chain complexes I, III, and IV has been closely associated with sperm motility (Ruiz-Pesini et al. 1998).

Unlike nDNA, mtDNA is not protected by histones, making it more susceptible to oxidative stress. Oxidative stress is measured by assessing malondialdehyde (MDA) levels in seminal fluid and ROS levels in sperm mtDNA. Elevated MDA and ROS levels have been observed in infertile males. Increased ROS and reduced antioxidant levels have been shown to negatively impact sperm count and motility, potentially leading to mtDNA mutations and impaired fertilization capacity of spermatozoa (Kumar et al. 2009).

mtDNA mutations are also believed to affect female fertility by compromising both ovarian primordial and mature follicles. The accumulation of point mutations may disrupt the oocyte’s NADH/NAD^+^ redox state, which plays a key role in preventing oxidative stress and maintaining antioxidant defense mechanisms (Blacker and Duchen 2016; Yang et al. 2020).

## Therapy for mitochondrial diseases

Mitochondrial diseases manifest when heteroplasmic mtDNA mutations exceed a threshold level, meaning the ratio of mutated to non-mutated mtDNA surpasses a critical limit. Therefore, one therapeutic strategy involves reducing the levels of mutant mtDNA below this threshold to prevent the biochemical effects associated with the mutations (Jackson et al. 2020).

One approach to achieving this is through selective inhibition of mutant mtDNA replication, allowing propagation of only wild-type mtDNA, thereby increasing its copy number. Antigenomic peptide nucleic acids (PNAs), which bind to mtDNA templates containing deletion breakpoints or single deletion mutations – but not to wild-type mtDNA – can be used in a replicationdependent manner to eliminate heteroplasmy. This has been demonstrated using an in vitro replication run-off assay (Taylor et al. 1997).

Parkin, a cytosolic E3 ligase, can translocate to depolarized mitochondria and induce their elimination via autophagy. Long-term expression of Parkin has been shown to restore wild-type mtDNA CN and cytochrome c oxidase activity by selectively removing mitochondria carrying deleterious COX1 mutations (Suen et al. 2010). A successful reduction of pathogenic mtDNA has been demonstrated in *Drosophila* muscle through autophagy stimulation, mitofusin suppression, and activation of the PINK1/Parkin pathway, providing a basis for potential human therapies (Kandul et al. 2016).

Mitochondrial transcription activator-like effector nucleases (mito-TALENs), which cleave pathogenic mtDNA mutations, can induce a permanent reduction in deletion or point mutations (Bacman et al. 2013). Similarly, mitochondrially targeted zinc finger nucleases can execute site-specific cleavage, generating double-strand breaks in mutant mtDNA to prevent its replication, thereby reducing the mutant DNA haplotype load and offering a therapeutic model (Gammage et al. 2014).

Another promising technique is mitochondrial gene therapy using MITO-Porter, a liposome-based delivery system capable of transporting macromolecules into mitochondria via membrane fusion. This method has been validated for delivering nucleic acids to mitochondria in affected cells (Yamada et al. 2008; Kawamura et al. 2020).

**Figure 4:**
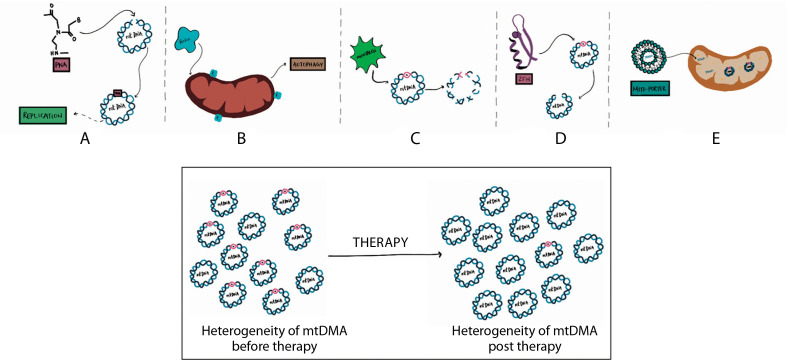
Therapeutic strategies for managing mitochondrial diseases. **A**) Use of antigenomic peptide nucleic acids (PNA) binds to deleted regions in mitochondrial DNA (mtDNA) and prevent their replication. **B**) The enzyme parkin eliminates depolarized mitochondria by autophagy. **C**) microTALENs cleave mutated DNA, facilitating their elimination. **D**) ZFNs sitespecifically cleave mutant mtDNA, aiding its reduction. **E**) MITO-Porter can deliver substituent nucleic acids to mitochondria that contain mutant mtDNA

Identification of mitochondrial mutations and the resulting conditions are important because they provide an understanding of the risk of developing the conditions in the presence of the mutation and an association between the diagnosis of the condition with the mutation, thereby making possible targeted treatments (Figure 4). Identifying the mutations can however only give a vague idea about the risk of developing the condition in healthy patients since the occurrence of heteroplasmy in cells cannot be entirely determined or managed.

Screening for mitochondrial mutations can be performed using DNA isolated from blood samples, employing techniques such as restriction fragment length polymorphism (RFLP) or direct sequencing of amplified mtDNA (Khan et al. 2015). The presence of the same mtDNA mutation affecting multiple organ systems may serve as a valuable diagnostic clue, offering insights into disease progression and prognosis. Recognizing patterns of multiorgan involvement can guide the selection of appropriate genetic tests and contribute to accurate diagnosis.

In the context of genetic counselling, identifying these mutations can aid in understanding inheritance patterns and clarifying potential risks to offspring, thereby supporting informed decision-making for affected individuals.

These therapeutic modalities enable the successful reduction of the mutant mtDNA, thereby reducing the heterogeneity of mtDNA and restoring the homogeneity of mtDNA, which then reduces the severity of the disease.

## Conclusions

Mutations and CN variations in the mitochondrial genome — and their resulting disorders — have been well documented in numerous studies. Depending on the function of the organ bearing a high mutation load, a variety of abnormalities specific to that organ can develop. Additionally, a single mitochondrial mutation may simultaneously disrupt the normal function of multiple organs. Mitochondrial mutations often than not seem to have an additive effect on the lethality of a disease, if not a causative effect, thereby making it understandable that the mutations need to be dealt with when coming up with the much-sought therapies.
